# The root canal system: a separate ecological niche?

**DOI:** 10.1080/20002297.2017.1325196

**Published:** 2017-06-01

**Authors:** Ilona Francisca Persoon

**Affiliations:** ^a^ Academic Centre for Dentistry Amsterdam (ACTA), University of Amsterdam and Vrije Universiteit Amsterdam, The Netherlands

## Abstract

Apical periodontitis is caused by microbial infection of the root canal system. Next generation sequencing on pulverized teeth was used to give insight into the complex microbial communities present in the intricate root canal system. Bacteriome profiling revealed an immense diversity. The most frequently identified genera were *Prevotella, Lactobacillus, Actinomyces, Fusobacterium* and *Atopobium*. Additionally, 57% of the teeth were positive for fungi, although fungal diversity was limited. *Candida* and *Malassezia* were the most frequently identified fungi. Interestingly, root segments that were positive for fungi had a significantly different bacteriome, since they contained more *Actinomyces, Bifidobacterium*, four different *Lactobacillus* OTUs, *Propionibacterium*, and *Streptococcus*. Further research should focus on the ecological conditions that lead to the co-occurrence of these acidogenic bacteria and fungi, and what effect the complexity of the microbiome might have on the course of the infection, the interaction with the host and the development of alternative treatment strategies.

